# Probiotics for Preventing Upper Respiratory Tract Infections in Adults: A Systematic Review and Meta-Analysis of Randomized Controlled Trials

**DOI:** 10.1155/2020/8734140

**Published:** 2020-10-26

**Authors:** Laodong Li, KangKang Hong, Qixiang Sun, Huan Xiao, Lejin Lai, Moyu Ming, Chaoqian Li

**Affiliations:** ^1^Department of Respiratory Medicine, The First Affiliated Hospital of Guangxi Medical University, Nanning, Guangxi 530021, China; ^2^Department of Respiratory Medicine, The Fourth Affiliated Hospital of Guangxi Medical University, Liuzhou, Guangxi 545005, China

## Abstract

**Background:**

Upper respiratory tract infections (URTIs) are common and burdensome infectious illness. Several trials have reported that probiotics can prevent URTIs in adults.

**Objectives:**

To evaluate the efficacy and safety of probiotics in the prevention of URTIs in adults.

**Methods:**

PubMed, Web of Science, Embase, and Cochrane Library were searched for reports published from database inception to May 14, 2020. Randomized controlled trials (RCTs) comparing probiotics with placebo for the prevention of URTIs in adults were included.

**Results:**

Six RCTs with 1551 participants were included. Compared with the placebo group, the probiotics intervention group significantly reduced the incidence of URTI episodes (RR: 0.77; 95% CI: 0.68 to 0.87; *P* < 0.0001; *I*^2^ = 26%), the episode rate of URTIs (rate ratio: 0.72; 95% CI: 0.60 to 0.86; *P* = 0.0002; *I*^2^ = 99%), and the mean duration of one episode of URTI (MD: −2.66; 95% CI: −4.79 to −0.54; *P* = 0.01; *I*^2^ = 80%). The adverse events of probiotics were mainly mild gastrointestinal symptoms. There were no significant differences in occurrence rate of adverse effects between probiotics intervention and placebo group (rate ratio: 1.01; 95% CI: 0.80 to 1.26; *P* = 0.96; *I*^2^ = 99%).

**Conclusion:**

Low-quality evidence provides support that probiotics have potential efficacy for preventing URTI episodes in adults. More trials are required to confirm this conclusion.

## 1. Introduction

Upper respiratory tract infections (URTIs) are the most common diseases, including the rhinitis, sinusitis, tonsillitis, pharyngitis, laryngitis, and common cold. Adults suffer from cold two to three times each year, and the global incidence of URTIs was estimated to be 17.2 billion in 2015 [1, 2]. URTIs are mainly caused by various viruses, such as influenza virus, adenovirus, rhinovirus, and respiratory syncytial virus [3]. Symptoms include stuffy nose, runny nose, cough, sore throat, headache, body aches, chills, fever, and so on. Most of URTIs are mild, but symptoms seriously affect the work and study of the infected individuals. URTIs are one of the most common reasons for seeking medical care and abusing antibiotics in some countries [4]. Therefore, it has great significance to prevent episodes of URTIs.

Probiotics are live microorganisms that promote health benefit when consumed in adequate amounts [5]. Studies have suggested that they possess the abilities of immunomodulation, intestinal epithelial barrier improvement, and pathogen inhibition [6–8]. In recent years, the effects of probiotics in the treatment and prevention of disease have been extensively studied by researchers [9]. Limited evidences showed that probiotics were beneficial for treating acute diarrhea [10], preventing eczema [11], and *Clostridium difficile* infection [12]. The effects of probiotics in the prevention of URTIs in adults remain controversial. The results of some randomized controlled trials (RCTs) have shown that probiotics can reduce the incidence, the number of episodes, severity, and duration of URTIs in adults [13, 14]. However, some studies reported that probiotics cannot reduce the number of URTI episodes [15, 16]. Thus, the aim of this systematic review and meta-analysis was to evaluate the effects of probiotics in the prevention of URTIs in adults.

## 2. Methods

### 2.1. Inclusion and Exclusion Criteria

Participants with the age from 18 to 65 years, RCTs comparing probiotics with placebo for the prevention of URTIs, were included. The probiotics included various strains, forms, and dosages. Studies were excluded for the following reasons: (1) they included other probiotics in placebo; (2) participants were vaccinated or took potential immune-enhancing dietary supplements during the trial process; (3) participants had congenital or acquired immune dysfunction; (4) participants had chronic allergies; and (5) participants took part in regular high-intensity physical exercise.

### 2.2. Outcome Assessment

The primary outcomes were the incidence of URTI episodes and the number of episodes of URTIs. Secondary outcomes included the mean duration of one episode of URTI and adverse events.

### 2.3. Search Strategy and Selection

A systematic search of PubMed, Web of Science, Embase, and Cochrane Library was performed for studies from database inception to 14 May 2020. Language was limited to English. The following search string was applied: (rhinit^*∗*^ OR sinusit^*∗*^ OR tonsillit^*∗*^ OR laryngit^*∗*^ OR pharyngit^*∗*^ OR “respiratory tract infection” OR “respiratory tract infections” OR “upper respiratory infection” OR “upper respiratory infections” OR “common cold” OR “common colds”) AND (probiot^*∗*^ OR prebiot^*∗*^ OR bifidobacterium^*∗*^ OR enterococ^*∗*^ OR Lactobacil^*∗*^ OR Lactococ^*∗*^ OR streptococ^*∗*^ OR saccharomyc^*∗*^) AND (random^*∗*^ OR placebo^*∗*^ OR crossover^*∗*^ OR “cross over” OR allocat^*∗*^ OR blind^*∗*^ OR Singl^*∗*^ OR doubl^*∗*^ OR trial^*∗*^). Duplicate articles were eliminated. We screened potentially eligible trials by titles and abstracts of articles obtained from the broad search, and then, full texts of these screened trials were assessed for eligibility according to the inclusion and exclusion criteria.

### 2.4. Data Extraction

The following data were extracted: the first author's name, year of publication, country, study design, study location, participants' characteristics, the number of participants in each group, probiotic strains, dosage, form, duration, the number of participants who experienced ≥1 URTI episode, the number of episodes of URTIs, the mean duration of one episode of URTI, and adverse events. All steps were performed independently by two researchers, and any disagreements were resolved by discussion with a third researcher.

### 2.5. Quality Assessment

The Cochrane risk of bias tool was used to assess the methodological quality of included trials. Two researchers evaluated each trial independently based on random sequence generation, allocation concealment, blinding of participants, blinding of outcome, incomplete outcome date, selective reporting, and other biases [17]. Discrepancies and divergence in the quality assessment were resolved by group discussion.

### 2.6. Statistical Analysis

In multiple arm trials, similar groups were combined to create a single pair-wise comparison according to the recommendations of Cochrane Handbook for Systematic Reviews of Intervention [18]. Dichotomous outcomes were expressed as risk ratio (RR) or rate ratio, and continuous outcomes were expressed as mean difference (MD), both with 95% confidence interval (CI). The rate ratio of the episode rate (the number of URTI episodes/person/year) of URTIs and adverse events rate between two groups and the standard error (SE) of rate ratio were calculated, and the generic inverse variance was used to pool these outcomes [4]. Statistical heterogeneity was assessed by using Cochran *Q* and *I*^2^ statistic. The *I*^2^ < 25%, 25–50%, and >50% were considered as low, mild, and substantial heterogeneity [19]. A random-effects model was used when the *P* value <0.05 or *I*^2^ ≥ 50%. In contrast, a fixed-effects model was used when the *P* value ≥0.05 and *I*^2^ < 50%. Sensitivity analyses were conducted by excluding each study individually to test the stability of the results. Data analyses were performed using RevMan version 5.3 provided by the Cochrane Collaboration, and *P* < 0.05 was considered statistically significant.

## 3. Results

### 3.1. Included Studies and Their Characteristics

**Figure 1:**
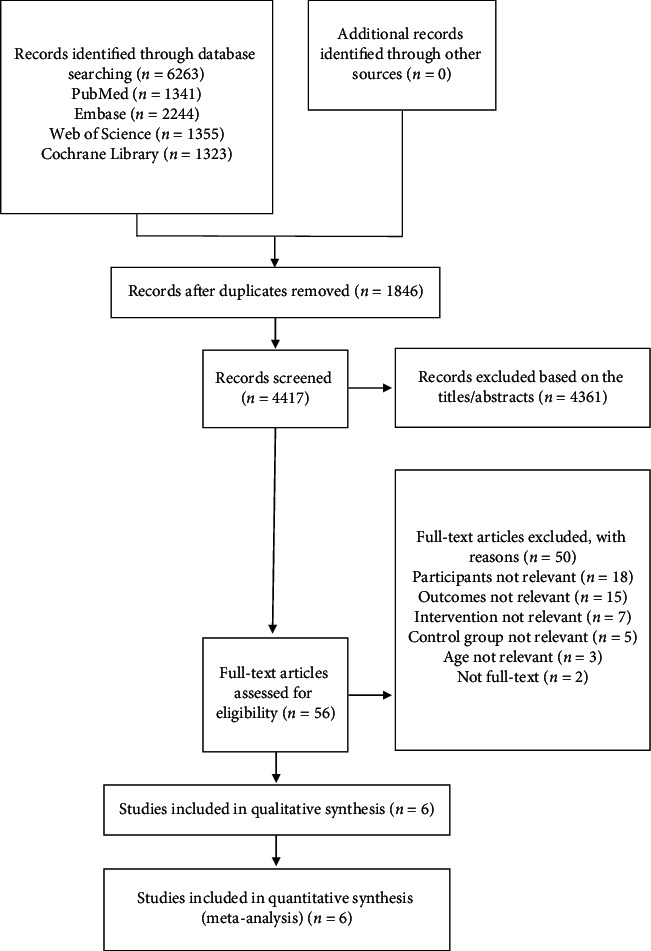
Flowchart of the literature screening.

The study flowchart is presented in Figure 1. A total of 6263 articles were identified by searching the databases, and 1846 duplicates were excluded. The remaining 4417 articles were screened by title and abstract, 4361 of which were excluded. We screened the remaining 56 articles carefully, and 6 articles [13, 15, 20–23] met our eligibility criteria and were ultimately included. The characteristics of included studies are presented in Table 1. These studies included 4 two-arm parallel [13, 15, 20, 21], placebo-controlled RCTs and 2 multiarm parallel [22, 23], placebo-controlled RCTs, in which one was a multicenter study [20] and the other five were single-center studies. In total, 1551 participants were involved, of whom 958 received probiotics and 593 received placebo.

### 3.2. Risk of Bias for the Included Studies

**Figure 2:**
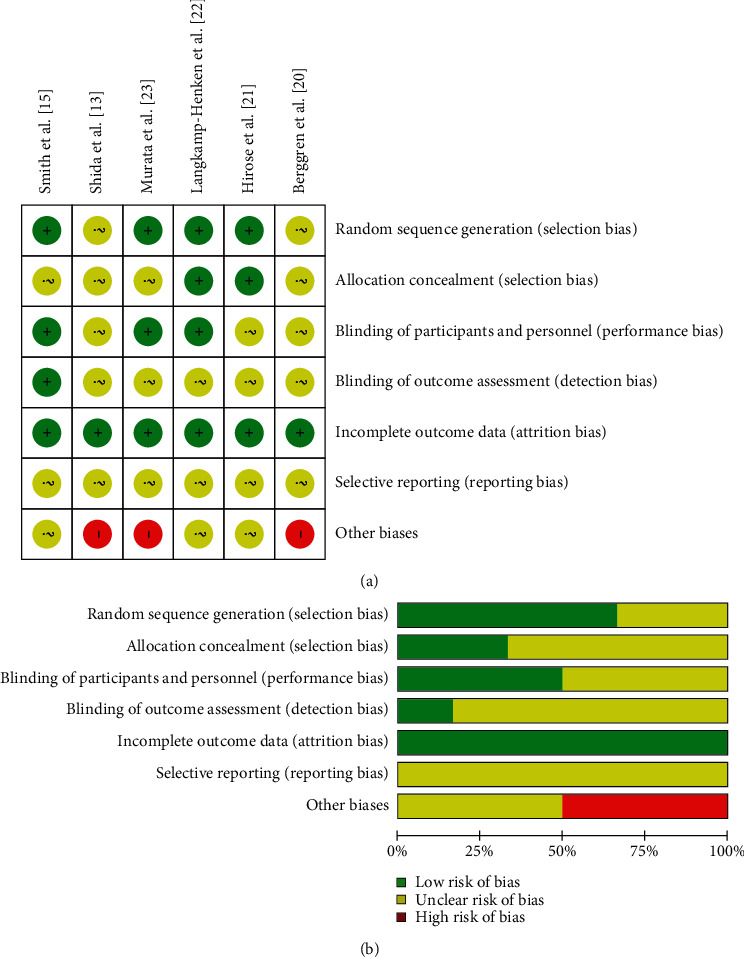
Risk of bias assessment for the included studies: (a) a summary for the risk of bias; (b) a graphic view for the risk of bias.

Four studies described adequate random sequence generation [15, 21–23], and only two studies reported adequate allocation concealment [21, 22]. Three studies did not clearly report the blinding procedure of participants and personnel [20–22], and five studies did not mention the blinding of outcome assessment [13, 20–23]. The participant dropout rates during follow-up were 0% to 16.2%, and all studies were judged as low risk of attrition rate. There was not enough information to assess the selective reporting in six studies. Three studies were judged as high risk for other biases because of the study's funding source [13, 20, 23]. The risk of bias for the included studies is shown in Figure 2.

### 3.3. Incidence of URTI Episodes

**Figure 3:**
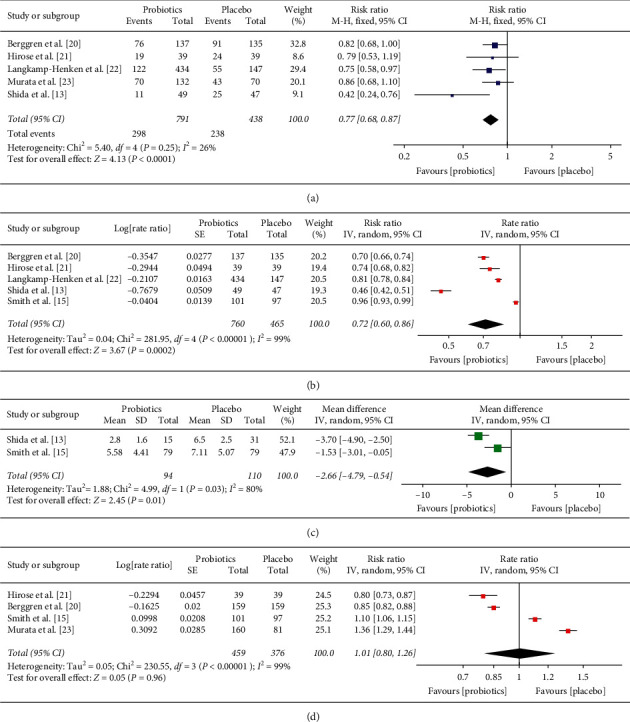
Forest plot of study results comparing probiotics with placebo groups: (a) the incidence of URTI episodes; (b) the rate ratio of episodes of URTIs; (c) the mean duration of one episode of URTI; (d) the rate ratio of the occurrence rate of adverse events.

Five studies reported the incidence of URTI episodes in the probiotic intervention group and placebo group [13, 20–23]. There were 791 participants in the probiotics intervention group and 438 participants in the placebo group. Pooled analyses showed that probiotics significantly reduced the incidence of URTI episodes compared with placebo (risk ratio: 0.77; 95% CI: 0.68–0.87; *P* < 0.0001; Figure 3(a)). A mild heterogeneity was observed (*I*^2^ = 26%; *P*=0.25).

### 3.4. The Number of Episodes of URTIs

Five studies reported the number of episodes of URTIs or the episode rate of URTIs [13, 15, 20–22]. There were 760 participants in the probiotics group and 465 participants in the placebo group. Pooled analyses showed that probiotics significantly reduced the episode rate of URTIs compared with placebo (rate ratio: 0.72; 95% CI: 0.60–0.86; *P*=0.0002; Figure 3(b)). However, there was substantial heterogeneity (*I*^2^ = 99%; *P* < 0.00001). We conducted sensitivity analyses by excluding each study individually. We found that this was not significantly different with the original analyses.

### 3.5. The Mean Duration of One Episode of URTI

Only two studies [13, 15] could be pooled with the mean duration of one episode of URTI because some studies reported the results as the mean duration of one participant's URTI episode. The result showed that probiotics significantly reduced the mean duration of one episode of URTI compared with placebo (MD: −2.66; 95% CI: −4.79 to −0.54; *P* = 0.01; Figure 3(c)). However, substantial heterogeneity was observed (*I*^2^ = 80%; *P* = 0.03).

### 3.6. The Adverse Events

Five studies reported adverse events, including nausea, vomiting, flatulence, abdominal pain, diarrhea, and bloating. Most of adverse events were mild [13, 15, 20, 21, 23]. Three studies suggested that none of the adverse events were associated with the trial intervention [13, 21, 23]. One study did not report the number of adverse events [13]. Four studies were pooled [15, 20, 21, 23], and the results showed that the occurrence rate of adverse events was not statistically different between two groups (rate ratio: 1.01; 95% CI: 0.80–1.26; *P*=0.96; Figure 3(d)). Substantial heterogeneity was observed (*I*^2^ = 99%; *P* < 0.00001), which was not significantly changed by using sensitivity analyses.

## 4. Discussion

It remains as an unsolved public health problem of finding effective prevention strategies for URTIs [24]. Recently, the number of studies that researched the potential effects of probiotics in the prevention of URTIs has increased dramatically. Previous meta-analysis showed that supplemental probiotics maybe a feasible strategy for preventing URTIs [4]. However, Hao et al. [4] included only three trials focused on adults [15, 20, 25], and most of the trials were conducted on children. Moreover, one of the included trials supplemented vitamins which were potentially immune-enhancing dietary supplements [25]. Our analysis specifically focused on adults and included recent updated RCTs.

We excluded participants who did regular high-intensity physical exercise because previous studies suggested that high-intensity physical exercise may affect immunity [26]. We also excluded participants who were vaccinated or took potential immune-enhancing dietary supplements during the trial process. In this study, only six RCTs were included according to our strict inclusion and exclusion criteria. The results in our meta-analysis indicated that probiotics could reduce the incidence of URTI episodes, the episode rate of URTIs, and the duration of one episode of URTI in adults. Furthermore, the occurrence rate of adverse effects in taking probiotics was not significantly different from taking placebo, and the adverse events of supplemental probiotics were mainly mild gastrointestinal symptoms. However, expect for the result of the incidence of URTI episodes, other results had substantial heterogeneity. Of note, these results did not significantly change during sensitivity analyses. Our results were consistent with previous findings on synbiotic, and Chan et al. [19] found that synbiotic could reduce the incidence and the episode rate of respiratory tract infections in adults.

The potential mechanism of probiotics in the prevention of URTIs is probably related to the modulation of the immune system. The study has shown that Lactobacillus casei Zhang can activate T-cells and B-cells and improve the levels of anti-inflammatory cytokines IL-4 and IL-10 [27]. Zhang et al. [28] reported that a combination of probiotics can increase the secretion of antiviral cytokines IFN-*γ* in blood and sIgA in the gut. In addition, natural killer (NK) cells play an important role in the prevention of URTIs. Probiotics could inhibit the reduction of NK cell activity and increase the level of salivary cortisol [13]. Besides, studies have suggested that intake of specific probiotics attenuated mental stress and reduced the risk of infection [23, 29].

This meta-analysis has some limitations. First, high heterogeneity was observed in our meta-analysis. The study population, probiotics strains, forms, and dosages varied among the included studies, which might be the reason for substantial heterogeneity. For example, we observed that the episode rate of URTIs in the B. bifidum R0071 group was lower than that in the L. helveticus R0052 group [22]. In addition, the diagnosis of URTIs was mainly through daily questionnaires, and participants might be misdiagnosed or missed diagnosis, which can affect the accuracy of the results. Third, the number of RCTs about probiotics compared with placebo for the prevention of URTIs in adults was relatively insufficient; therefore, it was not possible to conduct a subgroup analysis with age or probiotics strains groups. Finally, all included studies were in English, and selective reporting and publication bias could not be ignored.

## 5. Conclusion

In summary, probiotic supplementation may be an effective strategy to prevent episodes of URTI in adults. However, the quality of the evidence is low because of publication bias and substantial heterogeneity. More high-quality RCTs are required to confirm this conclusion and to assess which species of probiotics, forms, and dosages is the most efficacious in preventing URTI episodes in adults.

## Figures and Tables

**Table 1:** Main characteristics of studies included in the meta-analysis.

Study author/year	Country	Study type	Participants	Age, years	No. of cases^*∗*^		Probiotics	Dose (CFU/d)	Administration form	Duration
					Probiotics	Placebo				
Berggren et al., 2011 [20]	Sweden	RCT, double-blind	Healthy adults	18–65	159	159	L. plantarum HEAL9 and L. paracasei 8700 : 2	1 × 10^9^	Powder	12 w
Murata et al., 2018 [23]	Japan	RCT, double-blind	Healthy females (most were students)	≥18	82	81	Heat-killed L. paracasei MCC1849	1 × 10^10^	Powder	12 w
					78	—	Heat-killed L. paracasei MCC1849	3 × 10^10^	Powder	12 w
Shida et al., 2017 [13]	Japan	RCT	Healthy males	30–49	50	50	L. casei Shirota LcS-FM	1 × 10^11^	Milk	12 w
Langkamp-Henken et al., 2015 [22]	USA	RCT, double-blind	Healthy students	≥18	146	147	L. helveticus R0052	3 × 10^9^	Capsule	6 w
					142	—	B. bifidum R0071	3 × 10^9^	Capsule	6 w
					148	—	B. infantis R0033	3 × 10^9^	Capsule	6 w
Hirose et al., 2013 [21]	Japan	RCT, double-blind	Healthy subjects with high mental pressure	40–64	39	39	Heat-killed L. plantarum L-137	NR	Tablet	12 w
Smith et al., 2013 [15]	USA	RCT, double-blind	Healthy students	18–25	114	117	B. animalis ssp. lactis BB-12 and L. rhamnosus LGG	>1 × 10^9^	Powder	12 w

^*∗*^The number of participants in an intention-to-treat population (all the participants who were randomized to their original group, regardless of whether or not they completed the study). RCT: randomized controlled trial; NR: not reported.

## Data Availability

The data used to support the findings of this study are included within the article.
